# Supplementary material for: Identification of photoperiod-induced specific miRNAs in the adrenal glands of Sunite sheep (Ovis aries)
Source: Front Vet Sci. 2022 Jul 22;9:888207. doi: 10.3389/fvets.2022.888207 (PMC9354845; doi:10.3389/fvets.2022.888207)
Supplement: Supplementary file 1 [file Data_Sheet_1.ZIP › Supplementary file/miRDeep2 file.zip!/miRDeep2 file/Know_Structure/LP42A1/oar-mir-654.pdf]

miRBase precursor : oar-mir-654
Total read count : 1
oar-miR-654-5p read count : 0
oar-miR-654-3p read count : 1
remaining reads : 0

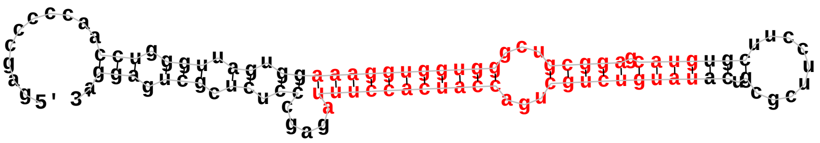

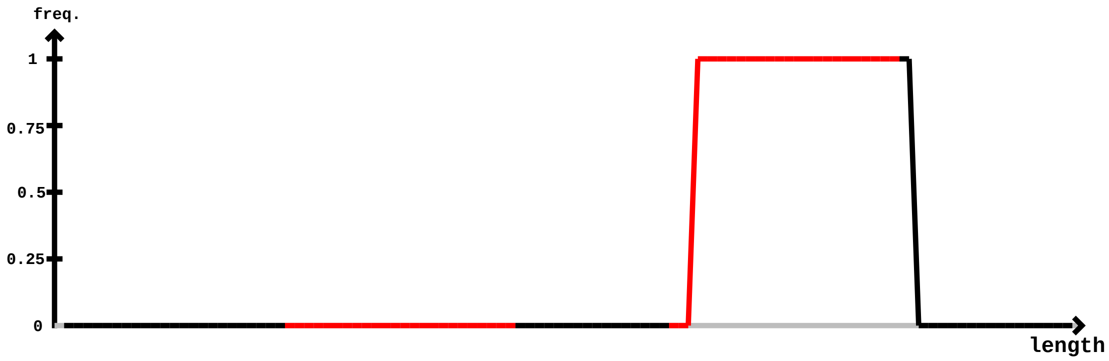

oar-miR-654-3p

oar-miR-654-5p

```
5'- gagcccccaaccuggguuaguggaaagguggugggcugcggagcaugugcuuccuucgcgucauaugucugcugaccaucaccuuuagagcccucucgcugagga   -3'    exp
    .(((((.......))))).((..(((((((((((...(((((.(((((((.........).)))))))))))...)))))))))))...))((((.....)))).    reads      mm         sample
    .................................................................ugucugcugaccaucaccuuuag.................    1          0          seq
```
